# Oral microbiota–host interaction: the chief culprit of alveolar bone resorption

**DOI:** 10.3389/fimmu.2024.1254516

**Published:** 2024-02-22

**Authors:** Jingyu Xu, Ling Yu, Surong Ye, Zitong Ye, Luyi Yang, Xiaoxi Xu

**Affiliations:** ^1^ Department of Orthodontics, Hospital of Stomatology, Jilin University, Changchun, China; ^2^ Key Laboratory of Dairy Science, Ministry of Education, College of Food Science, Northeast Agricultural University, Harbin, China

**Keywords:** oral host-microbial interactome, oral-systemic axis, periodontitis, *Porphyromonas gingivalis*, *Fusobacterium nucleatum*, bacterial extracellular vesicles, alveolar bone resorption

## Abstract

There exists a bidirectional relationship between oral health and general well-being, with an imbalance in oral symbiotic flora posing a threat to overall human health. Disruptions in the commensal flora can lead to oral diseases, while systemic illnesses can also impact the oral cavity, resulting in the development of oral diseases and disorders. *Porphyromonas gingivalis* and *Fusobacterium nucleatum*, known as pathogenic bacteria associated with periodontitis, play a crucial role in linking periodontitis to accompanying systemic diseases. In periodontal tissues, these bacteria, along with their virulence factors, can excessively activate the host immune system through local diffusion, lymphatic circulation, and blood transmission. This immune response disruption contributes to an imbalance in osteoimmune mechanisms, alveolar bone resorption, and potential systemic inflammation. To restore local homeostasis, a deeper understanding of microbiota–host interactions and the immune network phenotype in local tissues is imperative. Defining the immune network phenotype in periodontal tissues offers a promising avenue for investigating the complex characteristics of oral plaque biofilms and exploring the potential relationship between periodontitis and associated systemic diseases. This review aims to provide an overview of the mechanisms underlying *Porphyromonas gingivalis*- and *Fusobacterium nucleatum*-induced alveolar bone resorption, as well as the immunophenotypes observed in host periodontal tissues during pathological conditions.

## Introduction

Oral health is an indispensable element of general health and well-being ensuring the fulfillment of basic daily human functions. However, according to the 2015 Global Burden of Disease (GBD) study, about 3.5 billion people worldwide suffer from oral conditions (1). The pronounced global prevalence and severity of oral diseases have sparked significant concern among the public. These progressive chronic clinical diseases affect the teeth and various tissues within the oral cavity. Dental caries, periodontal diseases, oral mucosal diseases, and oral cancer are the main types of oral diseases, exhibiting high prevalence and severe adverse prognosis for individuals, communities, and society (2).

Beyond their prevalence and public concern, oral diseases are believed to have bidirectional associations with systemic health (3–7). Simultaneous or sequential occurrences of oral diseases and systemic diseases (8–19), such as gastrointestinal, immune, cardiovascular, and nervous system diseases, have been reported. Moreover, the tight relationship between human microbial communities and human health has drawn significant interest from researchers, with the oral microbiome considered to play a vital role in oral diseases and the connection between oral and general well-being.

Oral pathogens colonize the surfaces of different habitats within the oral cavity and form functional groups with pathogenic roles. Typical representatives of these groups include *Porphyromonas gingivalis* and *Fusobacterium nucleatum*. These two periodontal pathogens can disrupt bone homeostasis by excessively activating host immune responses. The resulting microbial–host interaction-induced local inflammation may spread throughout the body, leading to systemic diseases. In this review, we elucidate the mechanisms behind *Porphyromonas gingivalis*-and *Fusobacterium nucleatum*-induced bone resorption, construct the immune defense phenotypes of the human body against the invasion of oral pathogenic microorganisms, and further explore the interaction between oral microbial communities and the host.

## Oral microbial ecological guilds and oral diseases

The oral cavity is an open system where microbes are ingested with every breath, meal, and drink, colonizing through close contact with other humans, animals, or the physical environment. It provides a habitat for microbes, with suitable temperature, humidity, and nutrition. Despite there being millions of microbial species on Earth, only approximately 760 have been identified as major oral residents (20). In typical oral ecology, there are only 296 species-level microbial taxa (21), which are collectively referred to as the human oral microbiota (22). Alongside planktonic forms, the oral microbiota tends to assemble into complex spatial structures and form symbiotic communities to adapt to environmental changes and maintain microbial community and host homeostasis.

### Oral microbial dysbiosis and its pathogenic pathway

Microbial dysbiosis is generally considered a state that mediates the associations between microbiota patterns and disease states (23). As the oral cavity is an open ecosystem, oral microbial homeostasis is often challenged by many factors, such as genetics, gender, habitat, age, diet, living habits, and environment. Long-term nongenetic factors may cause genetic variation, resulting in dramatic changes in the structure of the bacterial flora (24).

The bidirectional association between oral microbial dysbiosis and general disease states might occur three distinct manners (25). Oral bacteria and their products can be transferred into the circulatory system via open or closed foci, such as inflammatory and ruptured epithelium or infection around the root apices. This transfer can cause transient bacteremia, resulting in systemic inflammation and metabolic and functional disorders (6, 26). Bacterial products, such as gingipains secreted by the typical periodontal pathogen *P. gingivalis*, have the potential to promote such pathological processes by degrading tight junction proteins, not only in periodontal tissues but also in vascular endothelial cells (27).

Oral pathogens can also be disseminated through non-hematogenous processes. Routes such as oro-pharyngeal or oro-digestive pathways may lead to ectopic colonization in the gut, disrupting the local microbial composition, triggering inflammation, compromising the intestinal mucosal barrier function, and inducing systemic diseases (28–31). An imbalance in gut homeostasis can promote the colonization of oral bacteria in the intestines (32–34). In addition, immune cells and factors responsive to oral pathobionts in the gut or other parts of the body can migrate to the oral cavity, exacerbating oral inflammatory conditions like periodontitis (35, 36). These processes illustrate the probability of an oral-systemic axis that regulates human health and disease conditions.

### Oral microbial niches and ecological guilds

Microbes in the oral cavity are not uniformly distributed. Only a few dozen species are abundant and constitute the core of the oral microbial community, whereas others are less abundant (37, 38). Heterogeneous colonization of oral microorganisms can be attributed to the uniqueness of oral niches, including the saliva, tongue, oral mucosa, mineralized tooth surfaces, and periodontal tissues (22). The spatial organization of oral microbes is in a state of dynamic equilibrium, maintained by opposing forces such as salivary flow, microbial adhesion, shedding and colonization, and crucially, microbe–microbe and microbe–host interactions (25, 39, 40). The microbiome colonizing the surface of mineralized teeth exists in the form of biofilms. Depending on their composition, nutritional background, ecological site anatomy, and antigen and immune exposure, plaques can be classified as subepithelial or subgingival (41, 42). Microorganisms within plaque biofilms rarely live independently; instead, they interact to form different functional groups and cooperate as ecological guilds to perform higher physiological functions (43). This applies to periodontal pathogens in subgingival plaques, where dominant species in the periodontal ecological guilds can determine the overall function of the group or play a crucial auxiliary role.

### Periodontal pathogens and bone remodeling

The role of periodontal pathogens in amplifying systemic inflammation and organ dysfunction has recently been established in several systemic diseases (44–46), such as inflammatory bowel disease (IBD), stroke, chronic renal diseases, cardiovascular diseases (47–49), diabetes (47), pneumonia, meningitis, rheumatoid arthritis (47, 50), cognitive disorders (51), as well as poor pregnancy outcomes (52, 53) and cancer (54). *Porphyromonas gingivalis*, a keystone periodontal pathogen, can ferment amino acids and grow deep in the glucose-poor periodontal pocket. *P. gingivalis* also invades gingival tissues and epithelial cells, promoting cell proliferation and causing epithelial radicular proliferation, which is a typical manifestation of periodontitis. The interaction between *P. gingivalis* and local host immune responses can have two contrasting outcomes, speculated to be related to the concentration of *P. gingivalis* and its virulence factors, mainly lipopolysaccharide (LPS), fimbriae, and gingipains. The concentration of virulence factors is high in the superficial layer, leading to immune escape, and low in the deep layer, resulting in a pro-inflammatory response that increases nutrient (heme) requirements (55). *P. gingivalis* employs unique and complex pathogenic mechanisms. These include strong invasive properties to allow it to enter the circulatory system, induce cell apoptosis, initiate oxidative stress, influence the host innate immune response by inducing dysfunction in neutrophils and macrophages, and facilitate the expression of acute phase proteins and numerous pro-inflammatory cytokines (52). Furthermore, *P. gingivalis* has the ability to regulate the innate immune response, ensuring the growth, colonization, and invasion of other opportunist and symbiont bacteria such as *F. nucleatum*, *Firmicutes*, *C. rectus*, *Streptococci*, *Staphylococci*, *Enterobacteriaceae*, *Prevotella*, *Hemophilus parainfluenza*, and *Dialister* (56–58). The dysbiotic microbiome induced by *P. gingivalis* is inherently resilient and can be stably transferred and easily restored even after antibiotic therapy is discontinued (59), making the local and systemic disease conditions triggered by *P. gingivalis* difficult to cure.

The obligate anaerobes *Fusobacterium nucleatum*, another core member of dental plaque, is believed to play a significant role in plaque maturation and dental plaque diversity (60). Its ability to co-cluster with various taxa serves as a physical bridge between early and late colonization of dental plaque organisms (60). Other hypotheses suggest that *F. nucleatum* acts as an indicator of establishing an anaerobic microenvironment and promoting plaque maturation (61–63), and has long been considered an initiating factor in periodontal disease. *F. nucleatum* tend to synergistically aggravate periodontitis and other systemic diseases when combined with *P. gingivalis* (52). However, despite being recognized as a periodontal pathogen, recent studies on *F. nucleatum* mostly discuss its role in tumorigenesis and immune evasion, with relatively few studies linking it to periodontal bone destruction.

The involvement of *P. gingivalis* and *F. nucleatum* in bone remodeling has always been a concern because of periodontitis. Periodontitis is a chronic inflammatory disease of the mouth that primarily develops from gingivitis. The accumulation of subgingival biofilm drives the progression from gingivitis to periodontitis, leading to the loss of periodontal supporting tissues. This progression occurs through continuous and complex interactions between the subgingival biofilm and the host’s immune response (21, 64, 65). Different clinical phenotypes of periodontitis have been associated with oral flora exhibiting different characteristics (66). While commensal gut microbes also have the capacity to regulate osteoimmune processes in the alveolar bone (67), *P. gingivalis* and *F. nucleatum*, which are oriented toward the commensal oral microbiota, have been shown to independently contribute to alveolar bone remodeling, separate from the systemic microbiome (39).

## Pathological mechanisms of alveolar bone resorption induced by periodontal pathogens

### Bone homeostasis in periodontal tissues

Pathogenic bacteria flourish in the gingival sulcus owing to their immune resistance, and their secretion of virulence factors or parasitic behavior can stimulate the immune response in the gingival tissues. This immune response effectively transmits virulence signals to the bone marrow cavity, leading to enhanced bone marrow hematopoiesis (39), which is an important pathway for immune cell generation. Under the dual stimulation of dysregulated bacterial flora and an excessive immune response, the homeostasis of alveolar bone tissue is unbalanced. To further demonstrate the roles of *P. gingivalis* and *F. nucleatum* in bone resorption, it is necessary to briefly review the mechanisms of osteoimmunology and the key regulatory axis of bone homeostasis, the receptor activator of nuclear factor-kappa B ligand (RANKL)–receptor activator of nuclear factor-kappa B (RANK)–osteoprotegerin (OPG) axis.

The term ‘osteoimmunology,’ coined by Arron and Choi in 2000 (68), refers to the field that investigates the interactions between immune cells and bone cells. These interactions mediate skeletal development, modification, and homeostasis under both physiological and pathophysiological conditions. Both innate and adaptive immune cells participate in bone turnover through direct contact or expressing a range of immune molecules, such as cytokines, chemokines, and immunoglobulins.

Recently, a research group provided a cellular atlas of specific oral mucosal positions in health and disease conditions, revealing a distinct stromal–immune responsive axis that dysregulates under inflammatory conditions. This axis may be capable of mediating periodontal osseous tissues homeostasis (69). The major cell types within healthy gingival tissues include epithelial cells, endothelial cells, fibroblasts, and immune cells. Within healthy gingival tissues, the immune category can be divided into five major clusters: T, NK, B/plasma, granulocyte, and myeloid cells, with T cells being the most numerous. T cells in gingival tissues can be subdivided into αβ CD4^+^T, TH17, mucosal-associated invariant T (MAIT), αβ CD8^+^T, γδ T, Treg, and NKT cells. The second largest population was myeloid linages, including neutrophils—which dominated this compartment—macrophages (Mφ), and myeloid dendritic cells (mDC). This result suggests that neutrophil-mediated innate immune responses are activated even when the periodontium is healthy. Sustained and highly coordinated neutrophil chemotaxis from the gingival vessels to the healthy gingival sulcus constitutes one of the major protective mechanisms against colonization by pathogenic microorganisms (65). Proper neutrophil monitoring targeting dental plaque biofilms has a dual benefit, conferring resistance to microbial colonization in periodontal tissues while maintaining an appropriate microbial composition for normal periodontal tissues function (70).

The epithelial and stromal cells present in the oral mucosa exhibit inflammation-related antimicrobial defense functions and can express transcriptional signatures of periodontitis inflammation and recruitment factors for neutrophils (69). This may be one of the reasons for the significantly elevated proportion of neutrophils in the oral mucosa. Stromal and immune cells can interact with each other through the expression of periodontitis susceptibility genes, becoming potential drivers of periodontal inflammation and immune cell over-recruitment, ultimately forming the basis of destructive hyperreactive immune responses (69).

Under healthy conditions, alveolar bone homeostasis is maintained by neutrophil-mediated innate immunity and T cell-mediated adaptive immunity. The cells and molecules involved stimulate bone remodeling cells, such as osteoblasts, osteoclasts, and their precursors, regulating their generation, development, function, and survival, ultimately maintaining bone homeostasis.

#### Osteoclasts and osteoblasts in bone homeostasis

Bone homeostasis is maintained by the coordinated action of mesenchymal-lineage-derived bone-forming osteoblasts and myeloid-lineage-derived bone-resorbing osteoclasts (71). Osteoclasts resorb osseous tissues by secreting hydrogen ions and lytic enzymes, while osteoblasts support mineralization by secreting unmineralized bone matrix and non-collagenous proteins (72).

Osteoclasts originate from monocyte–macrophage precursor cells, which are originally differentiated from HSCs. Studies have demonstrated that M1 macrophages contribute to osteoclastogenesis (73–75) under pathogenesis, and immature dendritic cells can develop into osteoclasts mediated by RANKL–RANK signaling (76, 77). Macrophage colony-stimulating factor (M-CSF) activates its cognate receptor c-Fms, inducing the expression of RANK on pre-osteoclasts (78), and consequently, induces the expression of NFATc1, a transcription factor that results in osteoclast proliferation and differentiation (79–82). Dendritic cell-specific transmembrane protein (DC-STAMP) (83, 84) and osteoclast stimulatory transmembrane protein (OC-STAMP) (85) are crucial for osteoclast maturation in a RANKL-dependent manner. RANKL-induced expression of the integrin-β3 subunit guarantees the αVβ3-mediated cell adhesion, which can seal certain podosomes, providing a critical microenvironment for osteoclast physiological functions such as motility and bone degradation/resorption (40–42). The secretion of cathepsin K, tartrate-resistant acid phosphatase (TRAP), and proteolytic enzymes occurs via the NFATc1-mediated RANKL signaling pathway (40–42).

Osteoblasts are mesenchymal lineage-originated osteogenic cells that eventually become bone-lining cells or osteocytes. Osteoblast differentiation and function are regulated by the transcription factors osterix and activating transcription factor 4 (ATF4), with the support of WNT, bone morphogenetic protein (BMP), fibroblast growth factor (FGF), insulin-like growth factor (IGF) signaling,. A recent study revealed that RANKL contributes to the osteogenic direction of bone marrow mesenchymal stromal cell (MSC) differentiation (86), indicating that membrane-bound RANKL, as a member of TNF superfamily, possesses the capability to act as a receptor for vesicular RANK derived from mature osteoclasts (87) or apoptotic bodies (88), performing reverse signaling from osteoclasts to osteoblasts and contributing to osteogenesis, and consequently promote the coupling of bone resorption and formation (87). This specific function is regarded as bidirectional signaling transportation, which might be closely related to the intracellular proline-rich motif (87). Given that membrane-bound RANKL is an easy-clustering-featured molecular and the clustering of this type of receptor was proven to induce cell activation (89–93), the accumulation and clustering of RANKL seems to be the critical mechanism triggering RANKL reverse signaling (94). Vesicular RANK binding to RANKL activates osteoblasts and promotes osteogenesis through mammalian target of rapamycin complex 1 (mTORC1) signaling and Runt-related transcription factor 2 (Runx2) activation (87). However, OPG, as a competing receptor for RANKL, cannot stimulate osteoblast activation owing to its characteristic of disturbing RANKL clustering (94).

**Figure 1:**
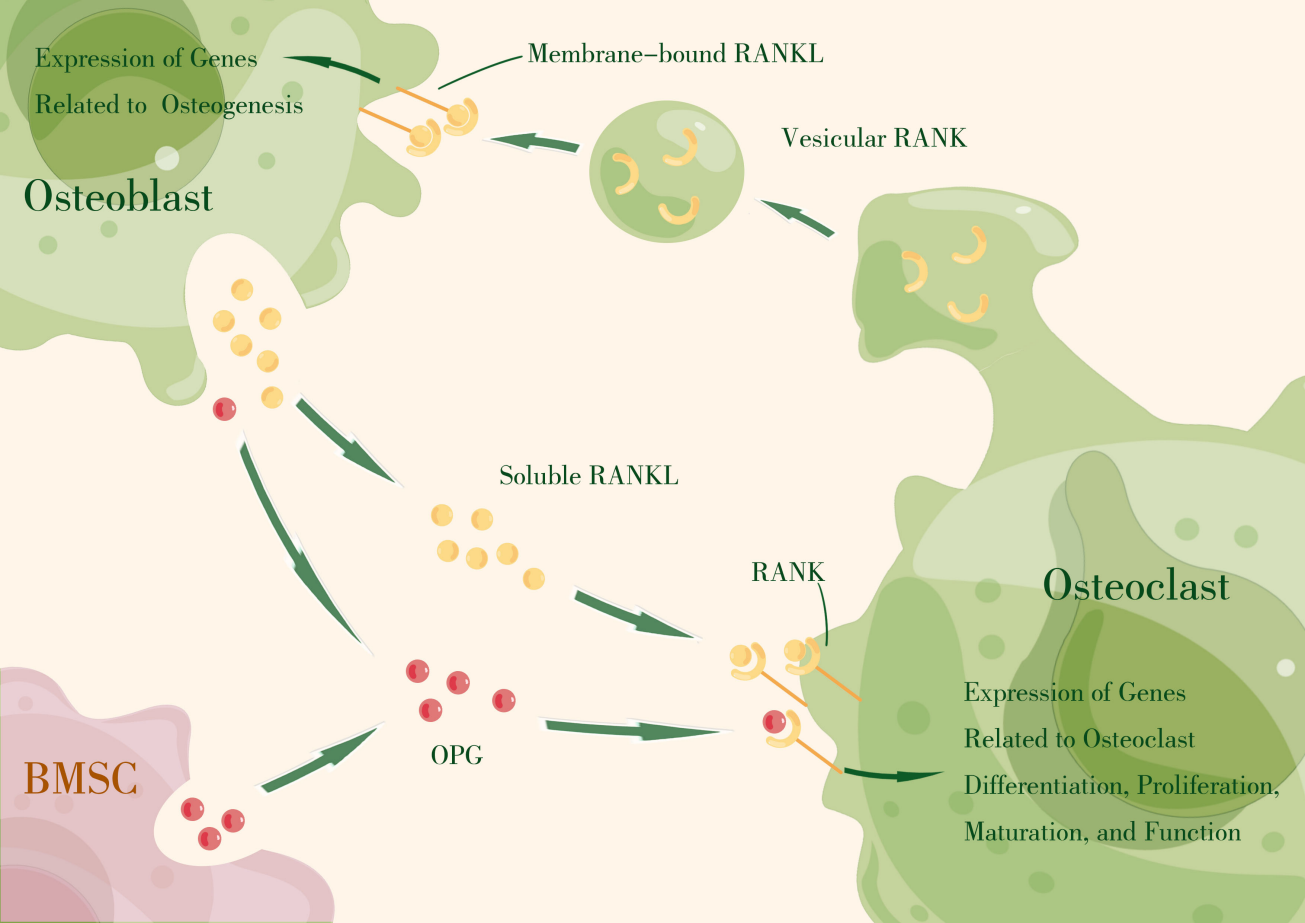
The RANKL–RANK axis produces essential signals that mediate intercellular communication in osteoclast–osteoblast coupling by regulating effector gene expression that drives cell proliferation, differentiation, maturation, function, and survival. OPG, primarily expressed by bone marrow stromal cells (BMSCs) and osteoblasts, acts as a decoy receptor, competitively binding RANKL to block RANKL–RANK interaction. (By Figdraw).

Thus, the RANKL–RANK–OPG axis produces essential signals that mediate intercellular communication in osteoclast–osteoblast coupling by regulating effector gene expression that drives cell proliferation, differentiation, maturation, function, and survival (**Figure 1**).

#### RANKL, RANK, and OPG

The receptor activator of nuclear factor-kappa B ligand (RANKL) and the receptor activator of nuclear factor-kappa B (RANK) were first discovered during the study of T-cell activation, and were found to be essential regulators of T cell and DC activation, thereby influencing T cell-mediated immune responses (95, 96). Subsequently, their critical role in osteoclast differentiation and bone remodeling was revealed (97, 98). RANKL, along with other biological mediators, regulates osteoclast differentiation, and under pathological conditions, it directly upregulates the expression of pro-osteoclastic cytokines and indirectly signals stromal-osteoblastic cells (99–101). Simultaneously, independent research groups identified RANKL as the osteoclast differentiation factor (ODF) from mouse myelo-monocytic cell lines and bone marrow-derived stromal cell lines (102, 103). Similarly, RANK was identified as the osteoclast differentiation factor receptor from mouse macrophage-like cell line (104, 105). Additionally, OPG was discovered to be an inhibitor of osteoclast differentiation (106, 107). These findings laid the foundation for understanding the regulatory effect of the RANKL–RANK–OPG axis in bone homeostasis.

RANKL, encoded by the tumor necrosis factor superfamily member 11 (TNFSF11) gene, is a type II homotrimeric membrane protein. It is produced by a variety of cell types, including osteoblasts, osteocytes, bone stromal cells, and immune cells within skeletal tissues. RANKL exists in three isoforms, with RANKL1 and RANKL2 being membrane-bound forms (108) that can be converted to soluble forms through proteolytic shedding (109, 110). RANKL3 lacks a transmembrane domain and is considered a soluble form (108). The membrane-bound form of RANKL can basically fulfill the function of this protein, but the soluble form contributes to physiological bone remodeling (111).

RANK, encoded by the tumor necrosis factor receptor superfamily member 11a (TNFRSF11A) gene, is a type I membrane receptor mainly expressed by hematopoietic cells, but also by osteoclasts and their precursors (78). It can also be detected on the surface of mesenchymal stem cells (86, 112). The intracellular domain of RANK contains a binding site for TNF receptor-associated factor (TRAFs) (113), which regulates the expression of genes associated with osteoclast function through the TRAF pathway (114).

OPG, encoded by the tumor necrosis factor receptor superfamily member 11b (TNFRSF11B) gene, is a member of the TNFR superfamily. It is primarily expressed by bone marrow stromal cells and osteoblasts, but can also be expressed in B cells, DCs, and follicular DCs. OPG exists only in its secreted molecular form and acts as a decoy receptor, competitively binding RANKL to block RANKL–RANK interaction (106). Local OPG is considered more crucial for skeletal and immune homeostasis compared to circulating OPG (115). In addition to RANK and OPG, LGR4 has been identified as a third competitive receptor that negatively regulates osteoclastogenesis through the GSK3-β signaling pathway by restraining NFATc1 expression (116). However, the binding affinity between RANKL and LGR4 is thought to be lower than that between RANKL and OPG, making OPG the main inhibitor of RANKL–RANK signaling (117).

The RANKL–RANK–OPG axis is a crucial signaling pathway for maintaining bone homeostasis through osteoblast-osteoclast coupling, with the concentration of soluble RANKL playing the key role. Disruptions in this pathway, caused by various stimulatory signals targeting RANKL secretion, can lead to an imbalance in bone homeostasis and contribute to pathogenic bacteria-induced bone resorption. In the following section, we will explore the virulence factors of major periodontal pathogens and their abilities to interfere with RANKL secretion through specific pathways.

### Virulence factors of periodontal pathogens and their pathogenic pathways

*P. gingivalis* and *F. nucleatum* possess various virulence factors that contribute to their pathogenicity. These factors play a significant role in the development and progression of periodontal disease. In recent years, there has been increased research interest in the role of bacterial extracellular vesicles (BEVs) in the pathogenic mechanisms of these microorganisms. We will explore these separately.

#### The virulence factors of *P. gingivalis*

*P. gingivalis*, an opportunistic pathogen and member of Socransky’s red complex, produces several virulence factors that induce detrimental effects on the host. The main virulence factors of *P. gingivalis* are LPS, fimbriae, and gingipains, which are crucial for the survival and metabolism of the bacterium.

LPS is an outer membrane component of gram-negative bacteria. It interacts with host cells, triggering a series of intracellular signaling events. LPS molecules consist of core polysaccharides, O-antigens, and lipid A; the latter two in *P. gingivalis* are highly diverse regions that confer antigenic differences and alter the interaction with pattern recognition receptors (PRRs), mainly TLR2, TLR4, and CD14. The disparity in LPS molecules depends on microenvironmental conditions (117) and sometimes leads to opposing immunological actions, immune evasion, or pro-inflammatory responses. This demonstrates that by manipulating the host immune activities, *P. gingivalis* can ensure its adaptation and survival (118, 119).

*P. gingivalis* LPS stimulates bone resorption in experimental models and activates various cell types, including mono-macrophages, endothelial cells, and epithelial cells, leading to the release of pro-inflammatory mediators and triggering immunoinflammatory reactions in the host tissues (120, 121). In vitro studies have also shown that *P. gingivalis* LPS increases the expression of pro-inflammatory cytokines in monocytes and macrophages, promoting bone resorption. In vivo, *P. gingivalis* LPS can activate mono-macrophages, endothelial cells, and epithelial cells through pathogen-associated molecular pattern (PAMP)-PRR recognition, resulting in the activation of cell signaling pathways like NF-kB and MAPK. These pathways ultimately stimulate the synthesis and release of IL-1, IL-6, TNF-α, NO, and other inflammatory mediators, contributing to a series of immunoinflammatory reactions in host tissues. In vitro, *P. gingivalis* LPS has also been proven to increase the expression of pro-inflammatory cytokines, such as IL-1, IL-6, IL-8, TNF-α, and IL-18, in monocytes and macrophages (122–125). These pro-inflammatory cytokines, including IL-1β, IL-6, and TNF-α, have been shown to stimulate bone remodeling cells and influence the RANKL–RANK–OPG axis, thereby promoting bone resorption.

Fimbriae are slender filamentous protrusions on the surface of *P. gingivalis* that that play a role in adherence and have pro-inflammatory capabilities (126–128). These fimbriae can stimulate signal generation through either TLR2 or TLR4, activating two distinct intercellular pathways. This activation leads to the production of pro-inflammatory factors and matrix metalloproteinases (MMPs), including TNF-α, IL-1, IL-6, IL-8, and MMP-9 (129, 130). Fimbriae also promote the expression of cell adhesins such as ICAM-1 (131). Moreover, fimbriae can interact with and activate the binding capacity of Complement Receptor 3 (CR3) through “inside-out” signaling (132, 133), facilitating the internalization of *P. gingivalis* by macrophages and reducing IL-12 production, which may inhibit bacterial clearance (133). Notably, fimbriae play a significant role in inducing bone destruction in experimental periodontitis models (134), and may be a target for immunotherapy aimed at reduce bone resorption (135, 136).

Gingipains, a series of cysteine proteinases generated by *P. gingivalis*, can be categorized into two types: arginine-specific (Arg-X) and lysine-specific (Lys-X) gingipains (137, 138). These gingipains can be present either on the cell surface or secreted in a soluble form. They are considered vital virulence factors of *P. gingivalis* but exhibit contradictory effects on innate immunity. On one hand, gingipains can activate protease-activated receptors (PARs) and act as pro-inflammatory stimulators and enhancers (139, 140) in neutrophils (141), gingival fibroblasts, gingival epithelial cells (142) and T-cells (143). They stimulate the production of IL-6 in oral epithelial cells (142) and IL-8 in gingival fibroblasts (144), and promote the recruitment of polymorphonuclear neutrophils (PMNs) through complement system activation (145, 146). On the other hand, gingipains can hinder the host immunity by cleaving several TCRs (147) and proteolytically inactivating factors such as IFN-γ, IL-4, IL-5, and IL-12 (148–151), even reducing bacterial opsonization (152) to cause increased resistance to bactericidal activity in *P. gingivalis*. Apart from manipulating host immunity, gingipains have also been shown to facilitate the adherence and invasion of fibroblasts and gingival epithelial cells (153–155), as well as increase vascular permeability and hemin availability in periodontal tissues, creating favorable conditions for *P. gingivalis* growth (156).

#### The virulence factors of *F. nucleatum*

*F. nucleatum*, a member of the Socransky’s orange complex, is a symbiont, opportunistic pathogen, and oncobacterium (157–159). Several virulence factors of *F. nucleatum* have been characterized, including FadA (160–164), which regulates adhesion and invasiveness; the heat-shock protein GroEL, which triggers host inflammatory factors (161); the endotoxin LPS, which activates NLRP3 and induces the release of inflammatory cytokines such as IL-1β (165); the metabolite butyric acid, which promotes the production of reactive oxygen species (ROS) and induces apoptosis of histocytes and immune cells (166); and multiple outer membrane adhesins (167) that can mediate the adhesion and coaggregation with various oral microbiota species, including *Streptococcus gordonii* (168), *Streptococcus sanguis* (169), *Streptococcus mutans* (170, 171), *Staphylococcus aureus* (172), *P. gingivalis* (173–177), and *Candida albican*s (178, 179). These virulence factors contribute to the expression of certain virulence factors, promote the formation and stability of plaque biofilm, and mediate the adhesion to immune cells (167).

*F. nucleatum* possesses various adhesins, which can be categorized into two types: amino acid inhibitors (e.g., RadD, CmpA, Aid1, FomA) associated with coaggregation with gram-positive bacteria, and lactose inhibitors (e.g., Fap2) associated with gram-negative bacteria. Coaggregation between *F. nucleatum* and *P. gingivalis* is mediated not only by a variety of adhesins but also by the capsular polysaccharide (CPS) and LPS, resulting in increased expression of virulence factors and altered energy metabolism in both species (180).

FadA is the most representative virulence factor of *F. nucleatum*, playing a crucial role in the adhesion and invasion of host cells. FadA exists in two forms: secretory and non-secretory. These two forms work together to regulate the adhesion and invasion of *F. nucleatum*. Through the interaction of the secretory autonomous transporter RadD and membrane occupation and recognition nexus protein 2 (MORN2) (181), *F. nucleatum* can invade gingival epithelial cells by binding to epithelial cadherin (E-cadherin). FadA can also help interact with the intracellular receptor retinoic acid-inducible gene I (RIG-I), activating the NF-κB signaling pathway to induce inflammatory responses and cause periodontal tissues destruction. Furthermore, *F. nucleatum* can promote epithelial–mesenchymal transition of gingival epithelial cells, up-regulating Snail-1 expression, down-regulating E-cadherin expression, and disrupting the integrity of the gingival epithelium. This promotes the invasion of pathogenic bacteria into deeper periodontal tissues (182). Recent research has discovered that *F. nucleatum* can secrete FadA-containing outer membrane vesicles (OMVs) which stimulate inflammatory bone loss in RA via the FadA–Rab5a–YB-1 axis in macrophages (183), and may have similar effects in periodontitis.

#### Bacterial extracellular vesicles

BEVs are spherical nanostructures encapsulated in bacterial lipid bilayers. They range in size from 20 to 300 nm and contain various functional active substances secreted by bacteria, including bacterial virulence factors and sRNA (184). Since the first discovery of extracellular vesicles in *Vibrio cholerae* in 1967 (185), BEVs have been considered an important mode of physiological and pathological functions in bacteria. They facilitate bidirectional communication between bacteria–bacteria and bacteria–cells, in addition to direct contact (186), and play crucial roles in bacterial colonization, survival, inflammation, pathogenesis, and regulation of host metabolism and immunity (187–194). At present, the field of cancer-related research believes that BEVs in the tumor microenvironment can be used as a new target for the diagnosis and monitoring of tumors and related diseases (195). Although research on BEVs in the oral pathological microenvironment is limited, these vesicles have the potential to provide valuable insights into the pathogenesis and pathological state of oral diseases, as well as the development of more efficient treatment methods.

#### Pathogenic pathways of virulence factors

The interaction between the oral microbiome (including living bacteria, virulence factors, and BEVs) and human immunity, known as the oral host–microbial interactome, promotes homeostasis under healthy conditions. The commensal microbiota educates and facilitates the immune system (196), imprinting innate and adaptive immunity memory to mount rapid and effective resistance against massive PAMP invasion. However, this immune memory can lead to overreactions and become a major cause of tissue destruction, including periodontal bone loss (6).

Studies have shown that dental biofilm plaque-induced bone loss in the periodontal tissues has an ‘effective radius of action’ known as the range of effectiveness. This range typically spans from 0.5 mm to 2.7 mm, with 2.5 mm being the precise measure (197–199). The constant distance between the base of the gingival groove and the alveolar crest, known as the biological width, is approximately 2 mm, falling within the range of effectiveness. This indicates that antigens and virulence factors present in biofilm plaque can traverse the epithelial barrier of the gingival tissues and penetrate the underlying connective tissues. Consequently, this triggers the release of paracrine signaling molecules, thereby affecting the balance of alveolar bone remodeling (65, 200). Research has demonstrated that the stimulation of PAMPs derived from subgingival plaque can elicit characteristic activation signals of bone marrow hematopoiesis, indicating the generation of immune cells derived from the myeloid lineage and the activation of associated immune responses (39). Meanwhile, innate immune cells present in the gums can uptake bacterial antigens from subgingival plaque and migrate to adjacent cervical lymph nodes, where they present antigens to activate the adaptive immune response. As a result, cytokines and immune cells, including T cells and memory T cells, may disseminate to the local gum tissues or even the entire body through the circulatory or lymphatic system (39, 201).

The oral microbial–host interactome can also transmit signals that extend beyond local tissues and contribute to the development of extra-oral comorbidities by initiating systemic inflammation or ectopic colonization in distant parts of the digestive tract (28, 36, 51, 196). Interestingly, a recent study suggested that the majority of healthy individuals do not exhibit detectable microbes in their blood, and even when a few species are detected, the microbial community patterns differ among various samples, with no apparent correlation between microbial species and the phenotype of healthy individuals (202). This implies that local disruption of the mucosal barrier serves as the initial step towards systemic comorbidities. Transient bacteremia facilitates the dissemination of microorganisms, such as oncobacteria, along with their virulence factors, to susceptible sites, thereby initiating or exacerbating disease progression at multiple sites. On a positive note, the microbial profile of gingival tissues in pathological conditions holds potential for aiding the diagnosis and treatment of extra-oral complications through blood microbial detection.

### Pathological osteoimmunity: activation of immune cells and cytokines

Under pathophysiological conditions, the subsets of immune cells that exist in a healthy state, such as T/NK, B/plasma, and granulocyte/myeloid cells, do not undergo significant changes in their overall categories. However, there are alterations in their proportions, particularly an increase in neutrophils and plasma cells (69).

The oral mucosal surface constantly faces microbial challenges, and neutrophils play a crucial role in maintaining alveolar bone homeostasis through innate immunity (203). Gingivitis is characterized by decreased neutrophils and bone activation factors, suggesting protective responses of the gingival tissues and bone during inflammation (66). However, as gingivitis progresses to periodontitis, there is an excessive inflammatory response leading to an increase in the number of neutrophils in local tissues. The quantity of neutrophils in the gingival tissues is more closely associated with the health or disease status of the periodontal tissues rather than their bactericidal function, which can be compensated by innate immune cells such as macrophages (204). Numerous studies have shown a positive correlation between the number of neutrophils in gingival tissues and the severity of periodontitis (205–207). In chronic periodontitis, dysfunctions in chemotactic accuracy, increased recruitment, and prolonged survival of neutrophils contribute to their extensive infiltration in periodontal tissues (204, 208, 209). These spontaneous hyperreactive neutrophils release various inflammatory factors (such as TNF, IL-1β, and IL-8), cytotoxic mediators, matrix metalloproteinases, and RANKL, which worsen periodontal tissues damage and bone resorption (210–213). Neutrophils can also migrate to the lymph nodes, where they interact with DCs to regulate antigen presentation and activate adaptive immunity (214). In the presence of CCL20, neutrophils can induce Th17 recruitment to inflamed tissues (215). They also promote B cell survival, proliferation, and differentiation into plasma cells by secreting B lymphocyte stimulator (BLyS) and a proliferation-inducing ligand (APRIL) (216, 217). Excessive neutrophils contribute to the progression of periodontitis and skeletal tissues destruction by initiating periodontal tissue lesions, exacerbating immune responses, and secreting local inflammatory factors and osteoclast-related factors. However, neutrophils deficiency in gingival tissues can also lead to periodontitis (218–220). Animal experiments have shown that impaired neutrophils recruitment associated with leukocyte adhesion deficiency Type I leads to increased periodontal inflammation, bone loss, and abnormal expression of IL-17 (221). This phenomenon might be related to a homeostasis mechanism of neutrophils recruitment, clearance, and generation, known as ‘neutrostat,’ which involves the IL-23–IL-17–granulocyte-colony stimulating factor (G-CSF) negative feedback loop (222). Impaired neutrophils recruitment results in unrestricted expression of IL-23, IL-17, and G-CSF in local tissues, leading to excessive inflammation and tissue damage (221).

Plasma cells are also significantly increased in patients with periodontitis compared to that in healthy individuals. The majority of plasma cells express IgG, while a minority express IgA (69). IgG is the main force in humoral immunity; it undergoes opsonization and antibody-dependent cell-mediated cytotoxicity (ADCC), and can activate the complement system through the classical pathway. These autoimmune responses may be the main factors contributing to periodontal destruction. Plasma cells may play a role in neutrophils recruitment by binding to the IgGFcR on the surface of neutrophils.

B cells have a dual role in periodontitis-related bone loss, which may depend on the activated B cell type. Certain B cell subsets exacerbate the severity of periodontal bone loss. In addition to IgG- and IgA-generated B cells, IgD- and IgM-generated B cells can also be associated with bacteria-induced periodontal bone loss, possibly through RANKL expression (223, 224). Memory B cells can promote osteoclast differentiation and maturation by expressing RANKL and various pro-inflammatory factors, such as TNF, IL-6 and IL-1β, and by increasing Th1 and Th17 production (225–228). Recent studies have highlighted the role of B cell activating factor (BAFF) in promoting periodontitis development by enhancing inflammatory conditions and macrophages activity (229). Conversely, regulatory B cells, also known as B10 cells, can reduce bone loss by upregulating IL-10 expression and downregulating IL-17 and RANKL expression (230–232).

In addition to neutrophils and B cells, T cells can be activated by antigens from *P. gingivalis* and *F. nucleatum* via TCR recognition and can differentiate into various subsets. Under pathologic conditions, T cells can affect bone remodeling by directly increasing the expression of pro-osteoclastic cytokines such as RANKL or indirectly signaling stromal–osteoblastic cells (99–101). Among all T cell subsets, γδ T cells (233); regulatory T cells (Treg) (234, 235); and helper T cells (Th, also known as CD4^+^T cells), including Th1 and Th2 (236, 237), Th9 and Th22 (238), Th17 (239), may be more closely associated with alveolar bone resorption (237).

Th1 and Th2 cells have been implicated in bone resorption in periodontitis, although the specific mechanisms have not been fully elucidated. The presence or absence of Th1 and Th2 cells may both contribute to bone resorption (240–243). The Th1/Th2 ratio was historically considered an important factor in evaluating the degree of bone resorption in periodontitis, as Th1 cells were believed to mediate the establishment of early periodontitis lesions, while Th2 cells gradually became quantitatively dominant as periodontitis progressed (236, 237). Interestingly, Th2 cells can promote the transformation of B cells into plasma cells by secreting IL-4, which may explain the increased proportion of plasma cells in gingival tissues under pathological conditions.

Current studies on bone remodeling have shifted focus from the Th1/Th2 balance to the Th17/Treg paradigm (67, 244, 245). Th17 has been closely associated with periodontitis and bone loss since their discovery (246, 247), and recent studies have shown that dysbiotic microbiomes activate Th17 cells to mediate oral mucosal immunopathology and periodontitis-induced bone destruction (239). Previous studies have demonstrated increased levels of Th17 and IL-17 in gingivitis and periodontitis (248–252), which are not necessarily related to the active or inactive stage of periodontitis (253–255). IL-23, a cytokine that promotes Th17 differentiation, is also highly expressed along with IL-17 in periodontitis (248, 253). Th17 cells, derived from naive T cells (also called Th0 cells) after stimulation by antigen presentation or pro-inflammatory factors (such as IL-1β, IL-6, and IL-23), are the primary source of IL-17 and can express other pro-inflammatory factors such as IL-21, IL-22, and TNF (256, 257). Although IL-17 may not directly act on the RANKL-macrophage colony-stimulating factor (M-CSF)-osteoclast culture system (258), it can promote osteoclastogenesis through the expression of RANKL mediated by osteoblastic cells (259). Interestingly, Th17-related neutrophil mobilization in gingival tissues can inhibit *P. gingivalis*-induced periodontal bone loss (260, 261), and IL-17 receptor α-deficient mice show reduced cytokine-dependent recruitment of neutrophils and increased bone resorption (262, 263), indicating that Th17 cells also possess bone-protective potential through neutrophil mobilization. Tregs, a subset of CD4^+^CD25^+^Foxp3^+^ T cells with anti-inflammatory and homeostatic functions, can secrete IL-10, IL-12, and TGF to achieve negative immune regulation. The presence of Tregs in periodontitis may represent a compensatory mechanism to mitigate excessive tissue damage caused by immune responses (237). Some studies have found that Tregs can improve pathological bone resorption through the CCR4–CCL22 pathway (234, 235). However, Tregs are highly plastic and can lose their immunosuppressive ability in chronic periodontitis (264). They may also differentiate into Th17 cells during the mid-stage of periodontitis (265). Therefore, the Th17/Treg ratio is a reasonable parameter to evaluate the dysbiotic microbiome-mediated periodontal inflammation status to a certain extent.

In addition to T cells, B cells, and neutrophils, other immune cells may also play a role in bone remodeling. NK cells in rheumatoid arthritis can promote osteoclastogenesis by expressing RANKL and M-CSF (266) and inhibiting osteoblast generation through a pro-apoptotic pathway (267). The degree of mast cell degranulation in chronic periodontitis is proportional to the severity of periodontal disease (268, 269), possibly owing to their ability to secrete IL-17 (270) and indirectly increase RANKL expression through IL-33 secretion (271). DCs synthesize and secrete a series of cytokines to increase RANKL expression (272–274) and immature DCs can differentiate into mature osteoclasts through the RANKL–RANK–M-CSF axis (76, 77, 274). Macrophages are recognized as immune cells that are closely related to osteoclasts. Macrophages are homologous to osteoclasts, as mentioned earlier, and in vivo, macrophages are able to participate in osteoclastogenesis through the RANKL–RANK–OPG axis, with the assistance of M-CSF.

## Concluding remarks and future perspectives

Oral health and systemic status are intertwined, with lesions in one affecting the other. Failure to address this cycle can lead to the progression of systemic diseases. The oral microbial community plays a crucial role in oral health, and any disruption in the ecological guilds can contribute to the development of oral diseases, including periodontal disease and the subsequent loss of periodontal hard tissues, which poses a significant threat to oral and systemic health.

**Figure 2:**
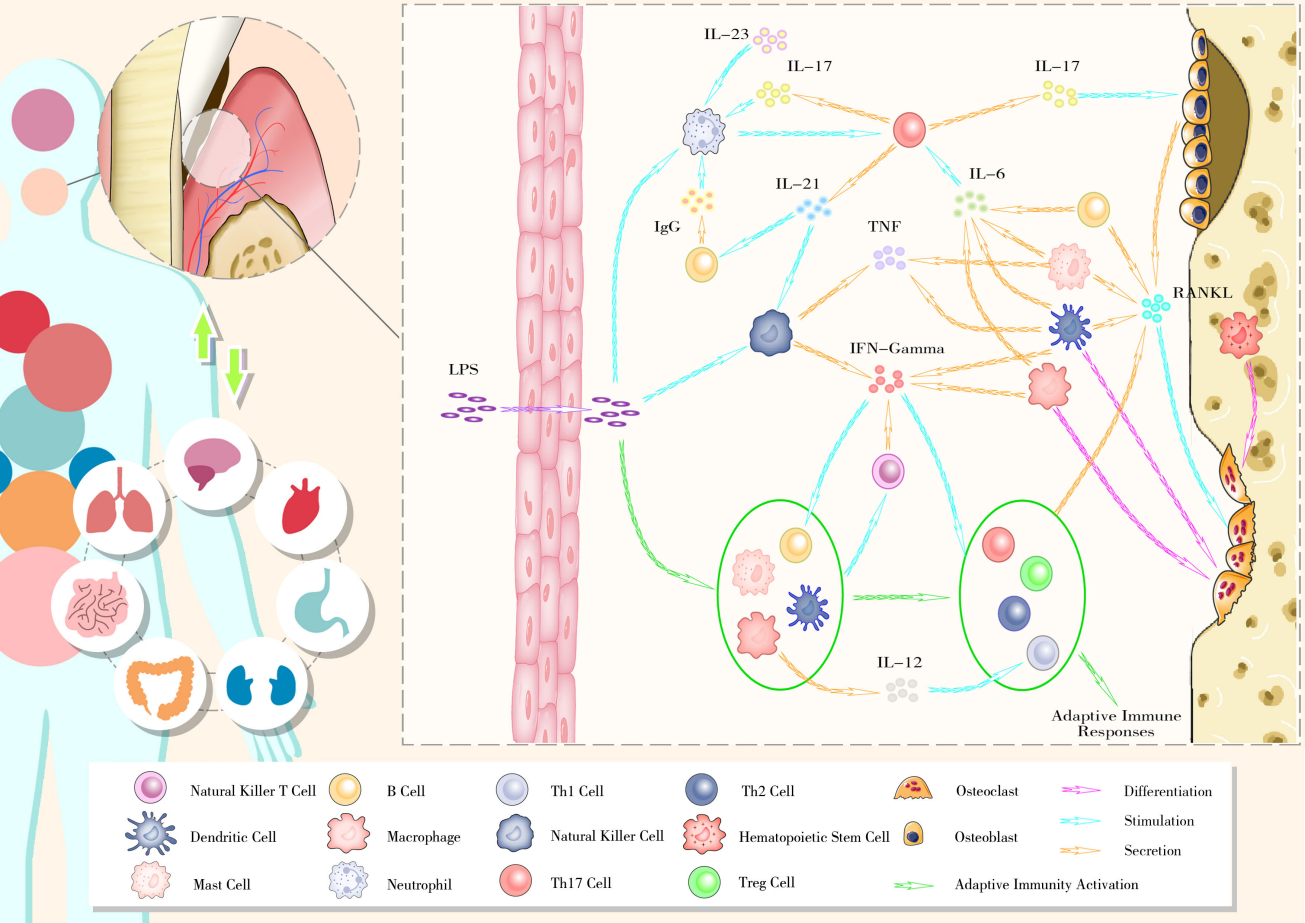
The bidirectional relationship between oral health and general well-being and the gingival immunophenotype of periodontitis.

The imbalance in osteoblast–osteoclast coupling, mediated by the RANKL–RANK–OPG axis, is at the core of alveolar bone remodeling disruption. Excessive immunity activation triggered by host–microbe interactions appears to be the primary reason for this imbalance. Key members in certain ecological guilds, such as *P. gingivalis* and *F. nucleatum*, drive periodontal inflammation with. Virulence factors from these pathogens activate the host immune system through local diffusion, lymphatic pathways, and blood transmission. Under chronic inflammatory conditions, continuous host–microbe interactions lead to an exaggerated immune response, resulting in periodontal tissues destruction and alveolar bone resorption (**Figure 2**).

Osteoimmunity involves intricate interactions between immune cells and molecules. The excessive osteoimmune response activated by the major functional microbiota associated with periodontitis cannot be solely attributed to changes in the proportion or function of individual immune cells. The phenotypes of the periodontal immune network must be established by studying the local and systemic immune status in the context of periodontal inflammation. Conversely, periodontal immunophenotypes reflect the characteristics of local ecological guilds. While the human body’s immunological characteristics are relatively clear and specific compared to the complexity of symbiotic microbial communities, the application of immunophenotypes holds promise as a straightforward method to evaluate the stability of plaque biofilms and study the symbiotic network of complex ecological guilds.

Although significant progress has been made in understanding the local immunophenotype of periodontitis and the role of pathogenic microorganisms, there are still gaps to be filled. Detailed investigations are needed to interpret the pathogenic effects of periodontal microorganisms. While the role of *P. gingivalis* in promoting alveolar bone resorption is well-established, there is limited research on the role of *F. nucleatum*, which has been recently focused as an oncobacterium in gastrointestinal tumors but not as a periodontal pathogen in alveolar bone resorption. Additionally, as the vital effect of extracellular vesicles gradually come into sight, the contents, secretion characteristics, and roles of *P. gingivalis* and *F. nucleatum* vesicles in bone remodeling are yet to be clarified. Furthermore, the contribution of other members within the periodontal pathogenic ecological guilds to alveolar bone resorption remains to be clarified.

Although the blood of healthy individuals is typically considered sterile, the presence of BEVs is a possibility. These vesicles may participate in immune system education in healthy individuals. However, once susceptible disease sites emerge, BEVs could potentially contribute to disease development even before the mucosal barrier is destroyed. Exploring the existence, content, and functions of vesicles in the blood of healthy individuals is an important area of investigation. Another aspect that remains to be elucidated is the immunophenotype of periodontitis. It is imperative to clarify the interaction network of immune cells and molecules in the disease state, identify the main functional groups, and screen characteristic high-expression cell phenotypes.

Maintenance of periodontal bone homeostasis is crucial in oral treatments that rely on physiological bone remodeling, such as periodontal therapy, orthodontic treatment, and implant restoration. How to block the progression of periodontitis, and restore lost bone, accelerate orthodontic effects by regulating bone remodeling, and how to reduce peri-implantitis to increase the success rate of implant surgery are all research focuses as well as difficulties in stomatology. However, these treatments often introduce various external stimuli to the teeth and periodontal tissues, resulting in oral hygiene challenges and disturbances to the periodontal microenvironment. To achieve optimal therapeutic outcomes, researchers should not simply focus on regulating the function of osteoblasts or osteoclasts, but aim to correct the unbalanced periodontal microenvironment and restore it to a healthy physiological state. By addressing these factors, some of the aforementioned clinical problems may find solutions.

Enabling patients to aesthetics and function healthfully is the fundamental principle of stomatology research. Modern medicine demands that dental practitioners not only control patients’ oral health during the short-term treatment and follow-up, but also maintain their lifelong well-being, which aligns with the WHO’s ‘8020’ goal, striving for improved oral health for the overall benefit of humanity.

## Author contributions

JX: Conceptualization, Visualization, Writing – original draft, Writing – review & editing. LiY: Resources, Writing – review & editing. SY: Resources, Writing – review & editing. ZY: Resources, Writing – review & editing. LuY: Funding acquisition, Supervision, Writing – review & editing. XX: Conceptualization, Supervision, Writing – review & editing.
